# Smallpox in Greece: A Narrative Review

**DOI:** 10.7759/cureus.97845

**Published:** 2025-11-26

**Authors:** Magdalini K Christodoulou

**Affiliations:** 1 School of Health Sciences, University of Thessaly, Larissa, GRC

**Keywords:** epidemics, greece, history of medicine, public health, smallpox, vaccination, variola virus

## Abstract

Smallpox was a highly contagious viral disease that represented a major cause of mortality prior to its global eradication in 1980. This narrative review explores the epidemiological, historical, and public health dimensions of smallpox in Greece from the 18th to the 20th century. It analyzes documented outbreaks, demographic impacts, and the evolution of disease control measures. Emphasis is placed on Greek physician contributions to early inoculation practices, implementation of vaccination programs, and legislative frameworks including the 1835 mandatory vaccination decree. The progression from reactive epidemic management to systematic preventive strategies through state-coordinated vaccination programs is documented, contextualizing Greece's experience within broader European public health developments. The review highlights how smallpox control efforts in Greece shaped the development of modern public health infrastructure and contributed to the broader global eradication campaign.

## Introduction and background

Smallpox, also known as variola, was a highly lethal infectious disease that affected human populations for centuries. Caused by the Variola virus, a member of the Poxviridae family, the disease was characterized by high mortality rates and severe complications among survivors, including blindness and permanent facial scarring [1]. It is estimated that in the 20th century alone, smallpox claimed the lives of 300-500 million people worldwide, making it one of the deadliest infectious diseases in modern history [2]. In Greece, smallpox represented a major public health challenge, particularly during the 19th century and the early decades of the 20th century. The newly founded Greek state, with virtually no health infrastructure and limited resources, faced frequent and severe epidemics. At the same time, Greek physicians of the 18th century, notably Emmanuel Timonis from Chios and Jacobus Pylarinos from Cephalonia, played a pioneering role in developing early prophylactic methods decades before Edward Jenner, who is traditionally credited with the discovery of vaccination [3]. Both physicians belonged to the Greek-speaking medical elite (Rums) of the Ottoman Empire, practicing in Constantinople and Smyrna, and bridging Eastern empirical medicine with European science. Timonis’s detailed account, published in Philosophical Transactions in 1714 (Vol. 29, pp. 72-82), and Pylarinos’s Nova et tuta variolas excitandi per transplantationem methodus (1715), described the practice of variolation already widespread in the Ottoman world [4]. The procedure later caught the attention of Lady Mary Wortley Montagu, who observed inoculations in Constantinople and introduced the practice to England in 1721 [5]. Their work thus represents the first documented scientific introduction of inoculation into Western medicine. This narrative review aims to examine the historical trajectory of smallpox control in Greece from the 18th to the 20th centuries, with particular emphasis on three interconnected dimensions: i) the evolution of public health policy from reactive epidemic management to systematic preventive strategies, ii) the pioneering contributions of Greek physicians Emmanuel Timoni and Jacob Pylarinos to early immunization science and their role in introducing variolation to Western medicine, and iii) the epidemiological and demographic impacts of smallpox on Greek populations during periods of political instability and nation-building. By contextualizing Greece's experience within broader European public health developments, this review demonstrates how systematic vaccination programs, initiated by the 1835 royal decree, transformed disease management and contributed to the global eradication campaign declared successful in 1980. The historical analysis draws lessons relevant to contemporary challenges in vaccine implementation, public health infrastructure development, and the management of vaccine hesitancy.

Methodology

This narrative review synthesizes historical, epidemiological, and public health evidence concerning smallpox in Greece from the 18th to the 20th centuries. Sources were identified through systematic searches of PubMed, Google Scholar, and specialized Greek medical history databases using keywords including "smallpox," "variola," "Greece," "vaccination," "Timoni," "Pylarinos," and "public health policy." Historical primary sources included Greek Government Gazettes (particularly the 1835 vaccination decree), archival medical society records, WHO historical documents, and contemporary medical literature from the Ottoman and Greek periods. Secondary sources encompassed peer-reviewed historical analyses, epidemiological studies, and recent molecular phylogenetic research published between 1714 and 2024. As a qualitative historical narrative review, this study does not employ quantitative meta-analysis, statistical pooling, or systematic risk-of-bias assessment tools typically used in clinical systematic reviews. Instead, the analysis integrates diverse source types, including legislative documents, clinical observations, demographic records, and molecular evidence, to construct a comprehensive historical narrative. The review prioritizes primary historical documents where available and contextualizes Greek developments within the broader European public health landscape. Selection of sources emphasized chronological coverage, geographic relevance to Greece and the broader Hellenic medical tradition, and scientific credibility of epidemiological claims.

## Review

Historical overview of smallpox

Antiquity and the Earliest Evidence

For many decades, the scientific community believed that smallpox was an ancient disease with a history spanning several millennia. This assumption was based mainly on historical descriptions of illnesses with symptoms resembling those of smallpox, as well as on archaeological findings, particularly facial scarring observed in ancient Egyptian mummies, notably the mummified body of Pharaoh Ramses V (ca. 1150 BCE). However, the identification of smallpox in such ancient remains controversial and unconfirmed at the molecular level and should therefore be interpreted with caution. Recent genetic studies have significantly reshaped our understanding of the virus’s evolutionary history [6]. In 2016, scientists successfully sequenced the complete genome of Variola virus from the remains of a mummified child who died around 1650 CE in Vilnius, Lithuania [7]. Phylogenetic analysis revealed that the virus is considerably younger than previously thought; rather than being thousands of years old, all known modern strains appear to share a common ancestor dating to the 16th-17th century [8]. This finding suggests that many ancient epidemics once attributed to smallpox may have been caused by other diseases with similar clinical presentations, such as measles or varicella [9]. Subsequent phylogenetic analyses have reinforced these findings, establishing a scientific consensus that Variola virus is considerably younger than previously believed, with implications for interpreting both ancient medical literature and archaeological evidence of pox-like disease.

Smallpox in the Roman Era and the Middle Ages

The Antonine Plague (165-180 CE), which devastated the Roman Empire, has been hypothesized by some historians to have been caused by smallpox [10]. During the time of Gregory of Tours (ca. 580 CE), a similar epidemic was recorded in France and Italy, where the term “smallpox” was reportedly used for the first time. Other scholars argue that smallpox was introduced into southwestern Europe by Arab armies returning from Africa during the 7th and 8th centuries. Throughout the Middle Ages, smallpox appeared sporadically in Europe but did not become firmly established until population growth and increased human mobility during the era of the Crusades facilitated its spread. By the 16th century, the disease had become endemic across the continent, with urban populations experiencing regular outbreaks [11].

*The 18th Century: The Age of Major Epidemics* 

Smallpox reached its zenith in Europe during the 18th century. It is estimated to have caused approximately 400,000 deaths annually, with a case fatality rate of about 30% [12]. Survivors often suffered severe long-term consequences: smallpox accounted for roughly one-third of all cases of blindness, while many bore permanent facial scars that marked them for life [13]. The disease spared no social class. During the 17th and 18th centuries, it claimed the lives of numerous European monarchs, including Joseph I, Queen Mary II of England, Peter II of Russia, and Louis XV of France. Others, such as Queen Elizabeth I and U.S. President Abraham Lincoln, contracted the disease but survived [14].

Clinical characteristics and forms of the disease

Pathophysiology and Transmission

Smallpox is caused by the Variola virus, a member of the genus Orthopoxvirus [15]. The virus is transmitted primarily via respiratory droplets expelled when an infected individual coughs or sneezes, but it can also spread through direct contact with contaminated objects, such as bedding or clothing [16]. The incubation period ranges from 7 to 17 days, with an average of approximately 12 days [17].

Clinical Presentation

The initial symptoms of smallpox resemble those of other viral infections and include high fever (often exceeding 38.5 °C), severe myalgia, malaise, headache, and profound systemic weakness. After 2-3 days, the characteristic rash appears, typically beginning in the oral mucosa before spreading to the face and extremities. The exanthem progresses over a period of 10-14 days through successive stages: maculopapular lesions, vesicles, pustules, and ultimately scabs, which often leave deep, permanent scars. Unlike varicella (chickenpox), all lesions in smallpox are at the same stage of development at any given time, a feature that historically aided differential diagnosis [18].

Clinical forms

Two primary clinical forms of smallpox were recognized [19]: Variola major, the most common and severe form, had a case fatality rate of up to 30% [20]. It was characterized by high fever, extensive rash, and, in its malignant and hemorrhagic variants, was almost invariably fatal. Variola minor, a less frequent and milder form, historically exhibited mortality rates of around 1%. This variant was first recognized in the United States and South America shortly before the end of the 19th century [21]. In addition, a third clinical presentation known as variola sine eruptione, or modified smallpox, as referred to in modern literature, was observed in vaccinated individuals. This form manifested primarily as fever without the typical exanthem [22].

The contribution of Greek physicians to the discovery of vaccination

The Pioneers: Timoni and Pylarinos

Although Edward Jenner (1749-1823) is traditionally regarded as the 'father of vaccination,' historical evidence indicates that the development of vaccination involved multiple contributors, including significant Greek contributions. Two Greek physicians of the 18th century, Emmanuel Timoni (1669-1720) from Chios and Jacob Pylarinos (1659-1718) from Kefalonia, were the first to introduce the practice of variolation into Western Europe in a systematic, scientific manner [23]. Both physicians had studied traditional medical practices observed among local healers (known as kolyges, elderly midwives or folk practitioners) in Thessaly and other regions of the Ottoman Empire. The method of variolation, well established in the Ottoman world, involved the deliberate inoculation of healthy individuals with material from mild cases of smallpox in order to induce protective immunity.

The Introduction of Variolation into Western Europe

Timoni, who studied at the Universities of Oxford and Padua, presented a detailed account of the variolation method practiced in Constantinople to the Royal Society of London in 1714. His report was published in the prestigious scientific journal Philosophical Transactions under the title “An Account, or History, of the Procuring the Small Pox by Incision, or Inoculation; as it has for some time been practised at Constantinople”. Meanwhile, Pylarinos, who also studied in Padua and served as a physician in Constantinople, conducted the first documented large-scale experimental inoculation in 1715 on three children, under the observation of multiple witnesses [24]. In 1721, with the support of Lady Mary Wortley Montagu, the wife of the British ambassador to the Ottoman Empire, variolation was officially introduced to Britain [25].

From Variolation to Vaccination

Despite its effectiveness, variolation carried significant risks: approximately 2-3% of those inoculated died from the procedure itself, and infected individuals could transmit the full-blown disease to others [26]. In 1796, Edward Jenner improved upon this technique by using material from cowpox (vaccinia) lesions to confer immunity [27]. This new method, known as vaccination, derived from the Latin vacca, meaning “cow”, was significantly safer and did not transmit smallpox. Jenner's work built upon the accumulated knowledge and experience derived from variolation practices and experience derived from variolation, a practice in which Greek physicians played a foundational role. The history of vaccination, therefore, should be understood as a collective scientific endeavor spanning multiple cultures, regions, and historical periods.

Smallpox in 19th-century Greece

Historical Context and the War of Independence

The 19th century opened with Greece still under Ottoman rule, but soon the country emerged as a small, independent state lacking any organized public health infrastructure. The Greek War of Independence (1821-1829) and the subsequent years of political instability created favorable conditions for the spread of infectious diseases.

Epidemics During the Revolutionary Period 

Between 1817 and 1819, outbreaks of plague struck Constantinople, Smyrna, and Crete. In 1822, plague was largely confined to northern Greece and to the Ottoman army advancing southward. In 1823-1824, Nauplion experienced an epidemic likely caused by a combination of typhus and smallpox. Among its victims was Benjamin of Lesbos, a prominent figure of the Modern Greek Enlightenment, who died there in 1824. From 1824 to 1829, a devastating smallpox epidemic that swept across Europe reached Constantinople, claiming numerous lives, including that of the eldest son of Sultan Mahmud II. When Ibrahim Pasha’s forces landed in the Peloponnese, they brought with them plague, which was endemic in Egypt at the time [28].

The Decree of King Otto (1835)

Following independence, the nascent Greek government sought to strengthen preventive medicine. On 11 May 1835, King Otto signed the decree “On the Introduction of Cowpox Vaccination” (Government Gazette No. 15), which marked the first organized public health intervention of the modern Greek state [29]. The decree established one of the earliest vaccination laws in Europe, contemporary to those of Bavaria (1807) and Sweden (1816), mandating cowpox vaccination for all newborns within the first year of life. Its provisions included the legal introduction of vaccination into Greek law, the mandatory immunization of infants, and the creation of a network of vaccination officers in each province. These officers were responsible for maintaining active vaccine material, conducting annual inspection tours, and providing vaccination services free of charge to all citizens, especially the indigent population. Non-compliance was punishable under Article 568 of the Penal Code. The decree established vaccination requirements for all newborns, created a network of provincial vaccination officers, and provided the framework for Greece's public health system. Following initial resistance from certain social groups, vaccination coverage increased progressively, resulting in reduced smallpox mortality during the late 19th century.

Epidemics in the Second Half of the 19th Century

Despite the introduction of vaccination, smallpox persisted as a major public health challenge throughout the 19th century. Vaccination coverage was uneven, with urban areas generally achieving higher immunization rates than rural or mountainous regions. Recurrent outbreaks were reported, particularly during wartime and periods of large-scale population movement. Alongside smallpox, Greece faced multiple severe epidemics during the century, including cholera, typhus, malaria, rabies, and tuberculosis [30]. The cholera epidemic of 1854 was particularly devastating. After arriving in Syros, the disease spread to Athens in October, triggering mass panic and population flight to surrounding villages. Approximately 20,000 residents, mostly the poorest, remained in the capital, and nearly 1,000 succumbed to the disease between October and December 1854 [31]. Other frequent infections of the period included childhood diseases such as measles, scarlet fever, and diphtheria, as well as recurring malaria and typhus epidemics, notably the one that struck Athens in 1894.

Development of Public Health Infrastructure

The repeated epidemics of the 19th century stimulated the gradual establishment of Greece’s public health infrastructure. The first hospital in Athens was founded in Piraeus in 1834, followed by a series of landmark institutions: the Ophthalmology Hospital (1854) under Andreas Anagnostakis, the Municipal Clinic (Astykliniki, 1857), the “Dromokaitio” Psychiatric Hospital (1887), the “Aretaieion” Hospital (1897), and the “Agia Sofia” Children’s Hospital (1900) [32]. The creation of lazarettos, quarantine and sanitary inspection stations, marked another important advance, improving epidemic containment and public hygiene.

Smallpox in the 20th century

The Early Decades

The 20th century began with periodic outbreaks of smallpox continuing to occur, although their frequency and severity had declined significantly thanks to expanding vaccination programs. In the early 1950s, an estimated 50 million cases still occurred globally each year. By 1967, the World Health Organization (WHO) estimated that 15 million people contracted the disease and 2 million died in that single year [33].

The Balkan Wars and World War I

The military conflicts of the Balkan Wars (1912-1913) and World War I (1914-1918) created conditions highly conducive to the spread of infectious diseases [34]. Soldiers and refugees facilitated the transmission of multiple pathogens, including smallpox. The so-called “trench diseases”, typhus, dysentery, and cholera, ravaged both troops and civilian populations.

The Interwar Period and the Asia Minor Catastrophe

The period between 1918 and 1940 was marked by significant efforts to improve public health infrastructure. A major dengue fever outbreak in 1927 overshadowed other infectious diseases during that era [35]. Smallpox, plague, and cholera were now typically confined to localized outbreaks with minimal mortality, thanks to widespread vaccination [36]. The Asia Minor Catastrophe of 1922 and the ensuing refugee crisis presented new public health challenges. Massive population movements were accompanied by outbreaks of infectious diseases, notably malaria, which emerged as the most pressing public health concern of the time [37].

The Lectures of the Medical Society of Athens

Scientific analyses of the lectures delivered by the Medical Society of Athens throughout the 20th century indicate that these presentations closely reflected contemporary public health priorities. Smallpox was frequently reported in the context of epidemic flare-ups but was associated with very low mortality. Vaccination and therapeutic strategies were central topics of discussion, and Greek physicians played an important role in disseminating international developments through their presentations [38].

Eradication of the Disease

The decisive breakthrough came in 1980, when the WHO declared the global eradication of smallpox. The last naturally occurring case was reported in Somalia in 1977 [39]. This historic achievement remains the first, and so far the only, instance of the complete eradication of an infectious disease through coordinated human intervention. In Greece, the last major smallpox epidemic occurred between 1910 and 1912, roughly seven decades before the official declaration of eradication [40]. The success of the national vaccination program, initiated by King Otto’s 1835 decree and progressively strengthened throughout the 20th century, was instrumental in protecting the Greek population from this deadly disease. Table 1 summarizes major events related to smallpox control, vaccination policy, and the evolution of public health infrastructure in Greece.

**Table 1:** Key Events in Smallpox Control and Public Health Development in Greece

Year/Period	Event	Impact
1714	Emmanuel Timoni publishes a description of variolation in Philosophical Transactions	First systematic scientific documentation of smallpox inoculation for Western medicine
1715	Jacob Pylarinos conducts experimental inoculation in Constantinople	First large-scale documented variolation trial observed by multiple witnesses
1796	Edward Jenner introduces cowpox vaccination	Safer and more effective alternative to variolation
1823–1824	Typhus and smallpox epidemic in Nauplion	Death of Benjamin of Lesbos; highlighted the need for organized public health measures
1835	Royal decree mandates compulsory cowpox vaccination (Government Gazette No. 15)	First organized state-led public health intervention in modern Greece
1854	Cholera epidemic in Athens (~1,000 deaths)	Stimulated development of public health infrastructure and sanitary reforms
1857–1900	Establishment of major hospitals	Founding of Municipal Clinic (1857), Aretaieion Hospital (1897), and Agia Sofia Children’s Hospital (1900)
1910–1912	Last major smallpox epidemic in Greece	Marked the beginning of sustained decline due to widespread vaccination
1977	Last naturally occurring smallpox case globally (Somalia)	Beginning of WHO eradication verification period
1980	WHO declares global eradication of smallpox	End of endemic smallpox transmission worldwide

The anti-vaccination movement: historical parallels

Early Reactions - Social and Political Context

Public mistrust and resistance to vaccination are by no means modern phenomena. As early as 1796, when Edward Jenner introduced his method of cowpox inoculation, the procedure was often met with skepticism and concern. A well-known caricature by James Gillray (1802) satirically depicted vaccinated individuals developing bovine features, reflecting widespread anxieties about the perceived unnaturalness of the procedure [41]. Opposition to vaccination in 19th-century Europe was deeply embedded in broader social and political tensions. Working-class communities frequently viewed mandatory vaccination laws as government overreach and class-based coercion, particularly when legislation imposed financial penalties on non-compliant families who could least afford them. In Britain, compulsory vaccination acts beginning in 1853 sparked significant social resistance, as impoverished parents faced imprisonment for refusing to vaccinate their children-a policy perceived as state intrusion into parental autonomy and bodily integrity. Religious objections were equally significant: many Christian denominations raised theological concerns that vaccination interfered with divine providence, while others regarded the procedure as an unnatural contamination of the human body with animal material. These objections were often amplified by genuine concerns about vaccine safety in an era when standardization and quality control were inconsistent, and adverse events, though rare, received considerable public attention. The convergence of class resentment, religious conviction, and legitimate safety concerns created a powerful opposition movement that would persist throughout the 19th century.

The British Anti-Vaccination Movement

In the century following Jenner’s discovery, opposition to vaccination in Britain evolved into a vast and organized social movement. The anti-vaccination campaign became intertwined with broader social unrest, religious objections, and anti-government sentiment. In 1898, the naturalist Alfred Russel Wallace famously denounced vaccination laws, claiming, as he provocatively stated, that ‘every day these laws remain in force, parents are punished and infants are killed. In reality, it was smallpox that claimed lives, an estimated 400,000 deaths annually in Europe during the 19th century, according to the World Health Organization. Vaccination remained the only effective preventive measure against a disease that was otherwise endemic and devastating [42].

Reactions in Greece

In Greece, King Otto’s 1835 decree mandating cowpox vaccination also encountered resistance, particularly from religious and conservative circles. Concerns were raised regarding the safety of the procedure, as well as theological objections that vaccination interfered with divine providence. Over time, however, improvements in public health indicators and the significant decline in smallpox mortality gradually persuaded the population of the vaccine’s effectiveness and safety. These patterns of resistance in Greece mirrored those observed across Europe, demonstrating that vaccine hesitancy transcended national boundaries and reflected common concerns about state authority, religious beliefs, and medical intervention. The gradual acceptance of vaccination in Greece, achieved through sustained public health education, demonstrable mortality reductions, and the visible success of immunization programs, offers historical precedent for addressing contemporary vaccine skepticism through evidence-based communication and transparent reporting of outcomes. These patterns of resistance in Greece mirrored those observed across Europe, demonstrating that vaccine hesitancy transcended national boundaries and reflected common concerns about state authority, religious beliefs, and medical intervention. The gradual acceptance of vaccination in Greece, achieved through sustained public health education, demonstrable mortality reductions, and the visible success of immunization programs, offers historical precedent for addressing contemporary vaccine skepticism through evidence-based communication and transparent reporting of outcomes.

The significance of historical experience for the modern era

Lessons From the Past

The history of smallpox and its eventual eradication offer valuable lessons for the present. It demonstrates that collective action, scientific research, and the coordinated implementation of public health measures can overcome even the most deadly infectious diseases. The eradication of smallpox remains one of the greatest achievements of medical science and international collaboration.

From Smallpox to COVID-19

The COVID-19 pandemic has revived historical parallels with past epidemics. As with smallpox, vaccines have once again proven to be the most powerful tool for disease control. Public skepticism and vaccine hesitancy observed during the COVID-19 crisis echo the resistance encountered during historical smallpox vaccination campaigns. However, there is also a fundamental difference: today we possess far greater scientific knowledge, technological capacity, and organizational resources than were available in the 19th or even the 20th century. The speed with which COVID-19 vaccines were developed reflects the remarkable advances in biomedical research.

The Value of Public Health 

The experience of smallpox underscores the importance of a well-organized and adequately funded public health system. The Greek state, beginning with King Otto’s 1835 decree, recognized early on its responsibility to protect public health and the critical role of compulsory vaccination as a preventive measure. Today, the need for robust public health systems is more urgent than ever. Globalization, human mobility, climate change, and other factors are creating new challenges and increasing the risk of emerging epidemics. The choice between investing in prevention and reacting to crises carries profound economic, social, and humanitarian consequences.

## Conclusions

Smallpox in Greece evolved from a recurring epidemic threat in the 19th century to a controlled and ultimately eradicated disease by 1980. The historical record demonstrates the progression from reactive epidemic management to systematic preventive strategies implemented through state-coordinated vaccination programs. Greek physicians Emmanuel Timoni and Jacob Pylarinos made significant contributions to the introduction of variolation practices into Western European medicine during the early 18th century, predating Jenner's vaccination method by several decades. The 1835 royal decree mandating compulsory vaccination represented one of the earliest governmental interventions in public health policy and laid the foundation for Greece’s modern public health system. The eradication of smallpox demonstrates the effectiveness of coordinated vaccination programs and international cooperation in infectious disease control. Historical evidence indicates that vaccine hesitancy is not a modern phenomenon but has accompanied immunization efforts since their inception. The scientific evidence accumulated over two centuries confirms that vaccination represents one of the most effective public health interventions for disease prevention. Contemporary public health challenges, including emerging infectious diseases and vaccine hesitancy, require continued investment in scientific research, public health infrastructure, and evidence-based disease prevention programs. The experience gained from smallpox eradication provides a framework for addressing current and future infectious disease threats through systematic vaccination strategies, international collaboration, and evidence-based public health policy.
